# A one-pot enzymatic approach to the *O*-fluoroglucoside of *N*-methylanthranilate^☆^

**DOI:** 10.1016/j.bmc.2013.05.057

**Published:** 2013-08-15

**Authors:** Lorenzo Caputi, Martin Rejzek, Thomas Louveau, Ellis C. O’Neill, Lionel Hill, Anne Osbourn, Robert A. Field

**Affiliations:** a Laboratory of Bioorganic Chemistry and Crystallography, Faculty of Science and Technology, Free University of Bolzano, Piazza Università 5, 39100 Bolzano, Italy; b Department of Biological Chemistry, John Innes Centre, Norwich Research Park, Norwich NR4 7UH, UK; c Department of Metabolic Biology, John Innes Centre, Norwich Research Park, Norwich NR4 7UH, UK

**Keywords:** Sugar nucleotide, Biotransformation, Fluorosugar, Glucosyltransferase, Natural product

## Abstract

In connection with prospective ^18^F-PET imaging studies, the potential for enzymatic synthesis of fluorine-labelled glycosides of small molecules was investigated. Approaches to the enzymatic synthesis of anomeric phosphates of d-*gluco*-configured fluorosugars proved ineffective. In contrast, starting in the d-*galacto* series and relying on the consecutive action of *Escherichia coli* galactokinase (GalK), galactose-1-phosphate uridylyltransferase (GalPUT), uridine-5′-diphosphogalactose 4-epimerase (GalE) and oat root glucosyltransferase (SAD10), a quick and effective synthesis of 6-deoxy-6-fluoro-d-glucosyl *N*-methylanthranilate ester was achieved.

## Graphical abstract

**Figure:**
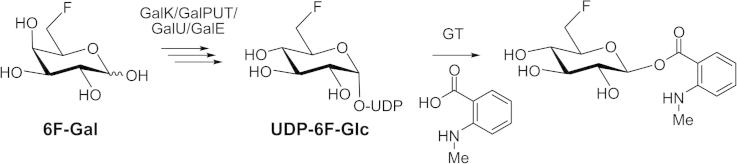

## 1 Introduction

Fluorine is an effective probe substituent that has found applications in a variety of different fields. Its chemical and physical properties make this element particularly interesting for NMR studies in organic and biological structural analysis and for bio-imaging techniques, such as magnetic resonance imaging (MRI) and positron emission tomography (PET), providing a wider range of chemical shifts and greater sensitivity when compared to hydrogen.^1^ In particular, ^18^F-PET has emerged as an important technique, not only for medical diagnosis and evaluation of treatment progress but also for understanding the mechanisms by which a biological activity is elicited. In this context, it represents a promising approach for imaging and quantitative assessment of the metabolic fate and accumulation sites of bioactive compounds, including drugs and nutraceuticals. ^18^F-PET enables non-invasive in vivo analysis without tissue destruction and without being influenced by the composition of the tissue studied.^2^ In this context, great effort has recently been devoted to the development of new and efficient strategies for the introduction of ^18^F atoms into peptides^3^, ^4^, ^5^, ^6^, ^7^, ^8^ and metabolites of interest.^9^, ^10^ The majority of the developed methodologies rely on the use of [^18^F]fluorosugars for labelling. Although the most widely used is 2-deoxy-2-[^18^F]fluoro-d-glucose, since is the common tracer for PET imaging and it is the primary source of ^18^F for nuclear medicine, other isomers of deoxy-fluoroglucose^11^, ^12^ as well as other fluorosugars,^13^ may be considered.

As the demand for new and effective strategies for introduction of ^18^F into tracers is increasing, the use of enzymatic routes is becoming more appealing due to the specificity of the reaction, leading to straightforward recovery of the desired products. Glycosyltransferases (GTs) are widespread enzymes in Nature with thousands of sequences already reported in different organisms (CAZy database).^14^ They catalyse the transfer of a sugar residue from an activated sugar donor, such as a nucleoside diphospho-sugar, to an acceptor. The acceptor substrates utilized by GTs are commonly other sugars, but can also be lipids, proteins, nucleic acids, antibiotics, or other small molecules. The glycosylation reaction can occur with –OH groups (most commonly) but it can also occur with –COOH, –NH_2_, –SH, and activated aromatic groups.^15^, ^16^, ^17^, ^18^ Some GTs are highly specific and are able to recognise only one or a limited range of acceptor substrates, whereas other GTs are promiscuous and can glycosylate a broad range of acceptors. GTs are considered useful synthetic tools for the preparation of natural oligosaccharides, glycoconjugates and their analogues;^19^ they are potentially interesting catalyst candidates for ^18^F radiochemistry, although their use may depend on the availability and suitability of an activated sugar donor.

Although fluorinated sugars have proved to be invaluable probes and inhibitors of glycosidases,^20^ the impact of sugar fluorination on glycosyltransferase-mediated glycosyl transfer is rather unpredictable. Effects of fluorination are dependent not only on the site of fluorine substitution but also on the type and source of the transferase in question. Before discussing the literature, a note of caution is required: the time windows and sugar nucleotide concentrations employed in some literature reports, particularly where fluorinated compounds are assessed as potential inhibitors, may not be compatible with forcing the turn-over of much less reactive fluorinated substrates. Hence reports of ‘no turn-over’ need to be considered in context. As would be expected, given the electronic impact of electron withdrawing fluorine adjacent to an acetal centre, sugar nucleotides bearing a fluorine atom at either the 2 or 5 position of the sugar ring do not generally serve as glycosyltransferase substrates, but they are effective competitive inhibitors of such enzymes.^21^, ^22^, ^23^ However, it has been reported that, in preparative biotransformations, GDP-2-deoxy-2-fluoro-l-fucose is indeed a substrate for FucTIII, although the same molecule is not a substrate for FucTVI.^24^ The metabolic incorporation of 4-deoxy-4-fluoro-GlcNAc into UDP-4-deoxy-4-fluoro-GlcNAc has been demonstrated in the human prostate cancer cell line PC-3. This leads to a metabolic block, since the fluorinated sugar-nucleotide is unable to transfer the fluorosugar to *N*-glycans, instead serving as a competitive inhibitor of natural sugar transfer and impacting on *N*-glycan profiles.^25^ GDP-6-fluoro-l-fucose is a competitive inhibitor of human fucosyltransferases III, V, VI and VII^21^ and recombinant porcine α-1,3-GalT and bovine milk β-1,4-GalT are unable to transfer 6-deoxy-6-fluoro-d-galactose onto an acceptor.^26^ In a similar vein, 6-deoxy-6-fluoro-d-glucose-1-phosphate is a very poor donor substrate for glycogen phosphorylase, displaying a 750,000 fold reduction in *V*_max_/*K*_m_ with respect to the parent glucose-1-phosphate.^27^ In contrast, the bacterial glycosyltransferase OleD (but not OleI or MGT) has been reported to transfer 6-deoxy-6-fluoro-d-galactose from the corresponding UDP-sugar adduct onto the glycosylated macrolide oleandomycin^28^ and, operating in the reverse sense, the OleD TDP-16 variant is able to produce UDP-6-deoxy-6-fluoro-d-galactose from the corresponding 2-chloro-4-nitro-phenyl glycoside.^29^ Further, UDP-5-deoxy-5-fluoro- and UDP-6-deoxy-6-fluoro-d-galactofuranose have been shown to serve as donor substrates and acceptor chain termination agents for Mycobacterial GlfT2.^30^

As is evident from this brief survey, the impact of donor substrate fluorination on glycosyltransferase-mediated glycosyl transfer is somewhat unpredictable. We were therefore minded to further investigate the potential of enzymatic synthesis to prepare sugar nucleotides substituted with fluorine, eventually settling on substrates substituted at the 6-position of the sugar to be transferred. The 6-fluorinated compound was also evaluated as a donor substrate for a representative glycosyltransferase, in this case the oat transferase SAD10, the physiological role of which is associated with the generation of an *O*-glucosyl *N*-methylanthranilate ester.^31^ Here we report an assessment of enzymatic approaches to 1-*O*-(*N*-methylanthraniloyl)-6-deoxy-6-fluoro-β-d-glucopyranose (6F-Glc-NMA). We report a convenient multi-enzyme biotransformation approach for generation of UDP-6-deoxy-6-fluoro-d-glucose (UDP-6F-Glc) and the subsequent glycosyltransferase-catalysed transfer of 6-deoxy-6-fluoro-d-glucose onto *N*-methylanthranilate. The strategy developed and the associated timeframes are compatible with ambitions to synthesise ^18^F-labelled materials for PET imaging studies.

## 2 Results and discussion

**Figure 1:**
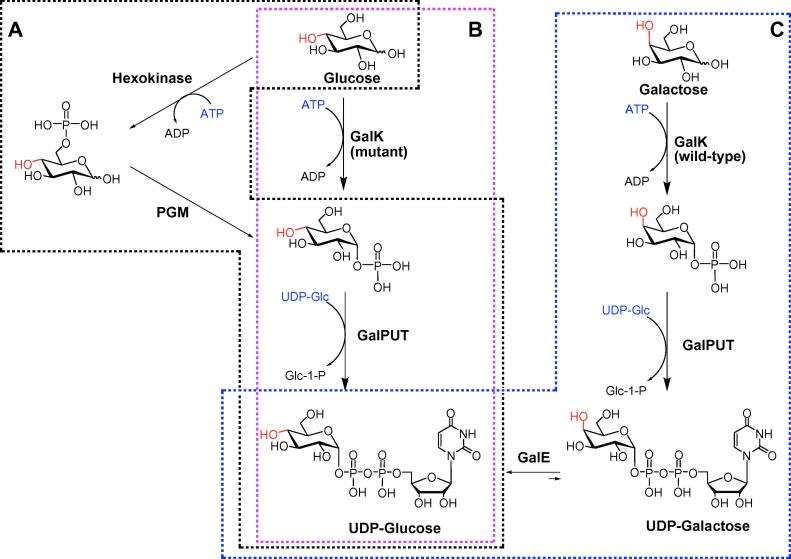
Outline of enzymatic approaches. (A) Hexokinase-phosphoglucomutase approach from Glc. (B) Mutant galactokinase-uridylyltransferase approach from Glc. (C) Galactokinase-uridylyltransferase-epimerase approach from Gal.

In order to investigate the ability of GTs to produce fluorine-labelled glycosides, we had a need to generate the corresponding fluorine-labelled UDP-sugar nucleotides. Taking into account the precedent for efficient C-2 fluorination via glycal chemistry,^32^ but the detrimental impact of installation of the electronic withdrawing fluorine adjacent to the anomeric centre, we surmised that ease and speed of fluorination at the hexose primary alcohol would represent a practical way forward. With this in mind, a number of enzymatic strategies were trialled (Fig. 1), with the aim of identifying the approaches that would be compatible with the short half-life of an ^18^F-labelled sugar (^18^F half-life 110 min).

### 2.1 The hexokinase-phosphoglucomutase approach

We initially explored the possibility of combining the phosphorylating activity of hexokinase to generate the glucose-6-phosphates (incompatible with the use of the 6-fluorosugar) with that of phosphoglucomutase, which transfers the phosphate from the 6-position to the 1-position. Subsequent action of galactose-1-phosphate uridylyltransferase would then give the desired sugar nucleotide (Fig. 1A). Employing the commercial yeast hexokinase and rabbit muscle phosphoglucomutase enzymes, this approach provided only very low conversion when starting from 3-deoxy-3-fluoro-d-glucose or 4-deoxy-4-fluoro-d-glucose. A similar approach has been reported by Prante et al.,^33^ when synthesizing UDP-2-deoxy-2-fluoro-d-glucose. However, the authors reported a conversion of 50% of the substrate in 6 days—far too long for applications involving the use of ^18^F. The ineffectiveness of the hexokinase-phosphoglucomutase approach could be ascribed to the fact that fluorosugars act as slow substrates for the hexokinase and phosphoglucomutase.

### 2.2 The mutant galactokinase-uridylyltransferase approach

A multi-enzyme method with recycling of the co-factors developed by Liu et al.,^34^ later modified by Errey et al.,^35^ starting from the reducing sugars was also evaluated (Fig. 1B). This method relies on two key enzymes, galactokinase (GalK) and galactose-1-phosphate uridylyltransferase (GalPUT). As shown before^35^ GalPUT possesses a broad substrate promiscuity. GalK, however, displays a high stringency for the C4-axial d-*galacto*-configuration of the sugar substrate, not recognizing d-glucose, for example. In a first set of experiments, the M173L-Y371H *Escherichia coli* GalK double mutant^36^ was investigated to directly phosphorylate fluorinated monosaccharides at the C1 position. This enzyme has previously been shown to possess relatively relaxed substrate recognition, allowing the direct anomeric phosphorylation of sugars with the C4-equatorial d-*gluco*-configuration. However, in our hands this approach was not effective in producing d-*gluco*-configured anomeric phosphates from glucose substituted with fluorine at the 3, 4 or 6 position, although the recombinant enzyme proved to be a competent galactokinase. In this instance perhaps the kinetic competence of the mutant is insufficient to support the efficient biotransformation of fluorinated d*-gluco*-configured sugars.

### 2.3 The galactokinase-uridylyltransferase-epimerase approach

**Figure 2:**
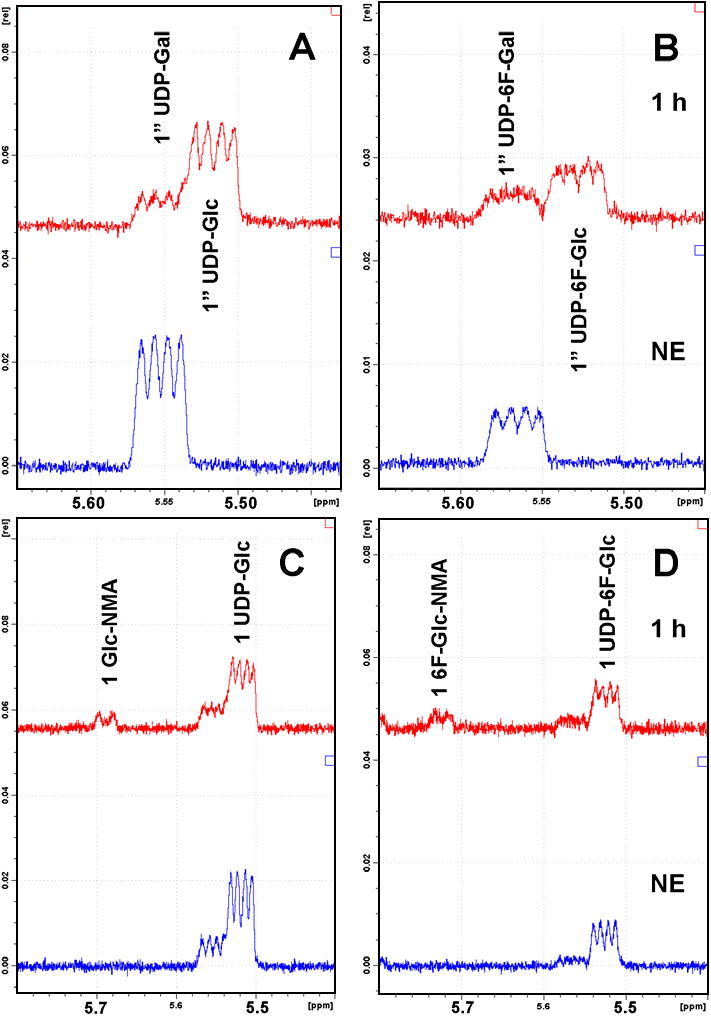
(A–D) ^1^H NMR signals of the anomeric protons H1″ and H1. (A) GalE mediated epimerisation of UDP-d-Gal into UDP-d-Glc. Lower trace, no enzyme (NE) control; upper trace, time point 1 h (UDP-Gal/UDP-Glc 1.0:3.8). (B) GalE mediated epimerisation of UDP-6F-d-Gal into UDP-6F-d-Glc. Lower trace, no enzyme; upper trace, time point 1 h (1.0:2.0). (C) SAD10 mediated transformation of UDP-d-Glc and *N*-methylanthranilic acid to give 1-*O*-(*N*-methylanthraniloyl)-β-d-glucopyranose (Glc-NMA). Lower trace, no enzyme; upper trace, time point 1 h (∼12% conversion). (D) SAD10 mediated transformation of UDP-6F-d-Glc and *N*-methylanthranilic acid to give 1-*O*-(*N*-methylanthraniloyl)-6-deoxy-6-fluoro-β-d-glucopyranose (6F-Glc-NMA). Lower trace, no enzyme; upper trace, time point 1 h (∼21% conversion). (C and D) The minor impurities (dd) are the residual *galacto*-configured sugar nucleotides starting materials.

**Figure 3:**
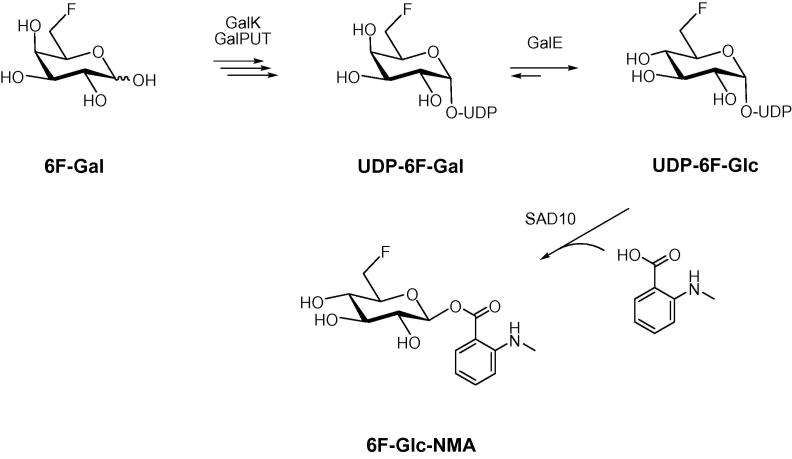
One-pot, multi-enzyme synthesis of 1-*O*-(*N*-methylanthraniloyl)-6-deoxy-6-fluoro-β-d-glucopyranose (6F-Glc-NMA).

Unable to achieve either the indirect or direct anomeric enzymatic phosphorylation of *gluco*-configured fluorosugars, we next approached the problem from a different angle (Fig. 1C). 6-Deoxy-6-fluoro-d-galactose, which we have previously shown to be a good substrate for wild-type GalK, was successfully converted into UDP-6-deoxy-6-fluoro-d-galactose (UDP-6F-d-Gal) (Fig. 3) following a multi-enzyme protocol, as reported previously.^35^, ^37^ This sugar nucleotide was then transformed into UDP-6F-d-Glc by the action of uridine-5′-diphosphogalactose 4-epimerase (GalE). This enzyme catalyzes the final step of the highly conserved Leloir pathway of galactose metabolism, epimerising the C4 position of UDP-galactose (UDP-Gal) to give UDP-glucose (UDP-Glc). GalE from galactose-adapted yeast was used and a control reaction, the epimerisation of UDP-d-Gal into UDP-d-Glc, was shown by ^1^H NMR (Fig. 2A) to progress rapidly until equilibrium was reached (UDP-Gal:UDP-Glc, ∼1:4). When UDP-6F-Gal was used as substrate, the enzyme retained its activity (Fig. 2B) and the equilibrium (reached in 3 h) of the reaction was strongly in favour of the *gluco*-configured product (UDP-6F-Gal:UDP-6F-Glc, ∼1:3), as in the control reaction.

### 2.4 Glycosyltransferase action with fluorinated and non-fluorinated donor substrates

In order to evaluate the potential of transferring the fluoro-sugar from UDP-6F-d-Glc onto a small molecule acceptor, we examined the plant glucosyltransferase SAD10.^31^ This GT1 family soluble enzyme is responsible for glycosylation of *N*-methylanthranilic acid using UDP-d-glucose as a sugar donor, which in our system was generated in situ from UDP-d-galactose using the epimerase GalE (Fig. 2A). The control experiment with the natural UDP-glucose substrate showed gradual formation of a broad doublet (5.69 ppm, 1H, bd, ^3^*J*_1,2_ = ∼6.8 Hz, H1) (Fig. 2C) indicating the formation of 1-*O*-(*N*-methylanthraniloyl)-β-d-glucopyranose (Glc-NMA) with conversion of about 12% at time point 60 min. The broadening of the anomeric signal is probably due to a restricted rotation along the Ar-COOR bond at ambient temperature. Extended incubation (20 h) resulted in equilibrium with a conversion of about 20%. Further experiments starting from Glc-NMA and monitoring the conversion to UDP-d-glucose and *N*-methylanthranilic acid showed that the equilibrium position for SAD10 lies at ca 30% Glc-NMA. These observations are in keeping with the ability of at least some glycosyltransferases to generate sugar nucleotides, not just to consume them.^36^, ^38^

Subsequent investigation of the SAD10-catalysed reaction of UDP-6F-d-Glc, generated above (Fig. 2B), and *N*-methylanthranilic acid was monitored by ^1^H NMR, showing the anomeric proton in UDP-6F-d-Glc (5.52 ppm, 1H, dd, ^3^*J*_1″,P_ = 6.8 Hz, ^3^*J*_1″,2″_ = 3.9 Hz, H1″) gradually becoming a set of two overlapping doublets (5.67 ppm, 0.5H, d, ^3^*J*_1,2_ = 5.6 Hz, H1 and 5.66 ppm, 0.5H, d, ^3^*J*_1,2_ = 5.8 Hz, H1) indicative of two rotamers of 1-*O*-(*N*-methylanthraniloyl)-6-deoxy-6-fluoro-β-d-glucopyranose (6F-Glc-NMA) (Fig. 2D). The conversion of UDP-6F-d-Glc into the product was about 21% at time point 60 min. Extended incubation (20 h) resulted in an equilibrium with conversion of about 31%, in keeping with that observed for the corresponding non-fluorinated substrate. An analytical sample of the product was purified by a combination of strong anion exchange chromatography (Poros HQ 50) and reverse phase HPLC (C18) and the compound was characterised by a combination of ^1^H and ^19^F NMR and LC–MS, the latter showing the expected molecular ion at *m*/*z* 316 ([M+H]^+^, 100%) and with characteristic fragment arising from sequential loss of water molecules (*m*/*z* 298, 280, 262) and liberation of *N*-methylanthranilate at *m*/*z* 152 ([NMA+H]^+^).

## 3 Conclusions

In summary, our efforts have identified a convenient galactokinase-uridylyltransferase-epimerase approach for the generation of UDP-6-deoxy-6-fluoro-d-glucose. Initial studies also confirmed that *O*-glucosyltransferase SAD10 is able to utilise this sugar nucleotide for the production of the fluoroglucosyl ester of *N*-methylanthranilate. Combining these observations, we were able to devise a two-stage, one-pot, multi-enzyme protocol that enables the rapid enzymatic synthesis of milligram quantities of the desired fluorine labelled natural product glycoside in a matter of minutes (Fig. 3). While the approach is dependent upon an expedient purification method, which we have demonstrated, the potential for the enzymatic generation of fluorosugar-containing small molecules in a timeframe compatible with ^18^F -labelling is clear.

## 4 Experimental

### 4.1 General methods

#### 4.1.1 NMR spectroscopy

NMR spectroscopy was performed on a Bruker Avance III spectrometer operating at 400 MHz (^1^H) or 376 MHz (^19^F). ^1^H signals were referenced to residual HDO at *δ*_H_ 4.70 ppm. Chemical shifts of ^1^H-decoupled ^19^F NMR signals recorded in D_2_O are reported with respect to external CFCl_3_ at *δ*_F_ 0 ppm.

#### 4.1.2 Chemicals

3-Deoxy-3-fluoro-d-glucose, 4-deoxy-4-fluoro-d-glucose, 6-deoxy-6-fluoro-d-glucose and 6-deoxy-6-fluoro-d-galactose were purchased from Carbosynth. Uridine-5′-triphosphate (UTP), phosphoenol pyruvate (PEP), adenosine-5′-triphosphate (ATP), and UDP-glucose were obtained from Sigma.

#### 4.1.3 Enzymes

Inorganic pyrophosphatase, pyruvate kinase, uridine-5′-diphosphogalactose 4-epimerase (GalE, from galactose-adapted yeast), yeast hexokinase and rabbit muscle phosphoglucomutase were obtained from Sigma–Aldrich. The *E. coli* GalK and GalPUT enzymes were over-expressed and purified as described in Errey et al.,^35^ Oat root glucosyltransferase SAD10 was cloned and over-expressed in *E. coli* as described in Owatworakit et al.^31^

### 4.2 Analytical and preparative HPLC-DAD

Analyses were performed on a Dionex Ultimate 3000 instrument equipped with a DAD detector. Analytical samples (50 μl) were collected at time points and quenched by addition of methanol (50 μl). The samples were centrifuged at 10,000 rpm and the supernatant was filtered through 0.2 μm disc filter. A sample (50 μl) was applied on a Poros HQ 50 strong anion-exchange column (L/D 50/10 mm, CV = 3.9 ml). The column was first eluted with 5 CV of 5 mM ammonium bicarbonate buffer, followed by a linear gradient of ammonium bicarbonate from 5 mM to 250 mM in 15 CV, then held for 5 CV, and finally back to 5 mM ammonium bicarbonate in 3 CV at a flow rate of 8 ml/min and detection with an on-line detector to monitor *A*_265_. After multiple injections, the column was washed with 3 CV of 1 M ammonium bicarbonate followed by 3 CV of MQ water. Further purification was performed on a Dionex Ultimate 3000 instrument equipped with a DAD detector. A solution of a sample in water was applied on a Phenomenex Luna 5 μm C18(2) column (L/D 250/10 mm, CV = 19.6 ml) and eluted with a gradient of acetonitrile against water at flow rate 5 ml/min: from 0% to 90% over 30 min then held for 2 min, then back to 0% acetonitrile over 3 min and equilibrated for 6 min. The on-line UV detector was used to monitor *A*_240_ and *A*_270_. Fractions containing the product were pooled and freeze-dried.

### 4.3 Enzymatic synthesis of UDP-6-deoxy-6-fluoro-d-galactose

The synthesis of UDP-6-deoxy-6-fluoro-galactose was performed using a multi-enzymatic reaction adapted from Errey et al.^35^ Briefly, 6-deoxy-6-fluoro-d-galactose (1 mg, 5.5 μmol), UTP (3.4 mg, 7.7 μmol), PEP (1.68 mg, 7.2 μmol), ATP (0.08 mg, 0.15 μmol) and UDP-glucose (0.094 mg, 0.15 μmol) were dissolved in 50 mM HEPES buffer (pH 8.0) containing 5 mM KCl and 10 mM MgCl_2_ (1 ml). After addition of GalK (10 μg), GalU (10 μg), GalPUT (10 μg), pyruvate kinase (2 U) and alkaline phosphatase (0.5 U) the reaction was incubated at 30 °C and monitored by HPLC-DAD (Poros HQ 50). After 2 h the reaction reached about 60% conversion. Methanol (1 ml) was added to quench the reaction and to precipitate protein. The mixture was centrifuged at 10,000 rpm, the supernatant was filtered through 0.2 μm PTFE filter and the product was isolated using HPLC-DAD (Poros HQ 50). Product containing fractions from multiple injections were pooled and freeze-dried to give the title compound as a diammonium salt (1.2 mg, 35%). Analytical data (^1^H NMR, ESI MS) were in agreement with literature.^35^

### 4.4 One-pot enzymatic synthesis of 1-*O*-(*N*-methylanthraniloyl)-6-deoxy-6-fluoro-β-d-glucopyranose

The enzymatic transformations were performed in a NMR tube in a total volume 700 μl. The reaction buffer (1 ml, 50 mM Tris–DCl, pD 7.8) was freeze-dried and re-dissolved in D_2_O (1 ml). UDP-6-deoxy-6-fluoro-d-galactose (1.0 mg, 1.6 μmol, final *c* = 2.4 mM) was dissolved in (690 μl) and the ^1^H NMR spectrum was acquired (no enzyme control). GalE (1 U, final *c* = 1.4 U/ml; enzyme in 10 μl of 100 mM HEPES buffer containing 100 mM NaCl, pH 7.0) was added and the progress of the epimerisation was monitored by ^1^H NMR spectroscopy. After 1 h the reaction reached approx. 67% conversion to UDP-6-fluoro-6-deoxy-d-glucose (further incubation to 20 h resulted in 82% conversion). Glucosyltransferase SAD10 (20.4 μg, final *c* = 28.7 μg/ml, enzyme in 10 μl of 100 mM Tris–HCl buffer containing 200 mM imidazole, 5% glycerol, and 300 mM NaCl, pH 7.5) was added along with *N*-methylanthranilic acid (242 μg, 1.6 μmol, final *c* = 2.3 mM) and the reaction was incubated at 30 °C. Formation of the glycoside product was monitored over the course of 20 h by ^1^H NMR (D_2_O, 400 MHz) spectroscopy following the diagnostic anomeric signals at *δ*_H_ 5.67 (0.5H, d, ^3^*J*_1,2_ = 5.6 Hz, H1) and 5.66 (0.5H, d, ^3^*J*_1,2_ = 5.8 Hz, H1). An analytical sample of the product was purified by a combination of strong anion exchange chromatography (Poros HQ 50) (peak eluting at the void volume of the column was collected) and reverse phase HPLC (C18) (product *R*_f_ = 16 min) and the title compound was obtained as a white solid after freeze-drying. ^1^H NMR (D_2_O, 400 MHz): *δ*_H_ 7.88 (1H, m, H_A_-aromatic), 7.42 (1H, m, H_C_-aromatic), 6.76 (1H, m, H_D_-aromatic), 6.60 (1H, m, H_B_-aromatic), 5.67 (0.5H, d, ^3^*J*_1,2_ = 5.6 Hz, H1), 5.66 (0.5H, d, ^3^*J*_1,2_ = 5.8 Hz, H1), 4.71–4.59 (2H, m, H6a, H6b), 3.81–3.52 (4H, m, H2, H3, H4, H5), 2.77 (3H, s, CH_3_). ^19^F NMR (D_2_O, 376 MHz): *δ*_F_ −235.9. LC–MS: peak eluting at *R*_f_ = 5.30 min. Full ESI-MS: *m*/*z* 316 ([M+H]^+^, 100%). MS2 of 316: *m*/*z* 298 ([M−H_2_O+H]^+^ 100%), 280 ([M−2H_2_O+H]^+^, 81%), 262 ([M−3H_2_O+H]^+^, 36%), 152 ([NMA+H]^+^, 18%), 134 ([NMA−OH]^+^, 28%).

### 4.5 HPLC–MS/MS confirmation of product formation

LC–MS analysis was performed on a Surveyor HPLC attached to a DecaXPplus MS (both Thermo). Separation was achieved on a 50 × 2 mm Luna 3 μ × C18(2) column (Phenomenex) using the following gradient of methanol versus 0.1% formic acid in water at 30 °C: from 5% to 90% then hold for 1 min at flow rate 0.3 ml/min then back to 5% methanol over 0.2 min and equilibrated for 2.3 min at flow rate 0.4 ml/min. Detection was performed by positive electrospray MS with spray-chamber conditions of 350 °C capillary temperature, 50 U sheath gas, 5 U aux gas, and a spray voltage of 3.8 kV using a steel needle kit. In addition to full MS from *m*/*z* 100–1500, the instrument was set up to collect data-dependent MS2 and MS3 of the most abundant precursor ions with collision energy of 35% and an isolation width of *m*/*z* 4.0. Dynamic exclusion was used to ensure that after two spectra had been acquired; the precursor would be ignored for 0.3 min in favor of the next most abundant signal.
